# Quinoxaline: *Z*′ = 1 form

**DOI:** 10.1107/S1600536810039905

**Published:** 2010-10-09

**Authors:** Sathishkumar Ranganathan, Sudarshan Mahapatra, Tejender S. Thakur, Gautam R. Desiraju

**Affiliations:** a Solid State and Structural Chemistry Unit, Indian Institute of Science, Bangalore 560 012, Karnataka, India

## Abstract

A new *Z*′ = 1 crystal structure of quinoxaline (or 1,4-diaza­naphthalene), C_8_H_6_N_2_, with one-fifth the volume of the earlier known *Z*′ = 5 structure was obtained by means of an *in situ* cryocrystallization technique.

## Related literature

For the structure of quinoxaline *Z*′ = 5, see: Anthony *et al.* (1998 ▶). For the crystal structure of the hydrated organic compound, see: Namba *et al.* (1981 ▶).
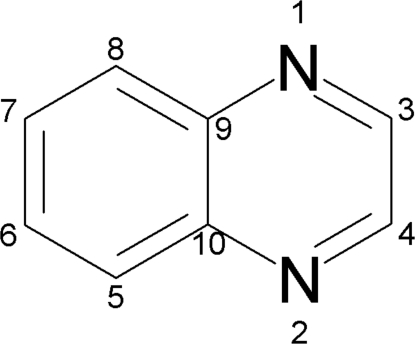


## Experimental

### 

#### Crystal data

C_8_H_6_N_2_
*M* *_r_* = 130.15
Orthorhombic, 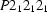

*a* = 4.0212 (13) Å
*b* = 7.187 (2) Å
*c* = 23.095 (7) Å
*V* = 667.5 (3) Å^3^
*Z* = 4
Mo *K*α radiation
μ = 0.08 mm^−1^
*T* = 270 K
0.40 × 0.30 × 0.30 mm

#### Data collection

Bruker SMART CCD area-detector diffractometer
Absorption correction: multi-scan (*SADABS*; Sheldrick, 1996 ▶) *T*_min_ = 0.968, *T*_max_ = 0.976
7556 measured reflections
956 independent reflections
494 reflections with *I* > 2σ(*I*)
*R*_int_ = 0.045

#### Refinement

*R*[*F* ^2^ > 2σ(*F* ^2^)] = 0.038
*wR*(*F* ^2^) = 0.106
*S* = 0.90
956 reflections
91 parameters
H-atom parameters constrained
Δρ_max_ = 0.13 e Å^−3^
Δρ_min_ = −0.15 e Å^−3^

### 

Data collection: *SMART* (Bruker, 1998 ▶); cell refinement: *SAINT* (Bruker, 1998 ▶); data reduction: *SAINT*; program(s) used to solve structure: *SHELXTL* (Sheldrick, 2008 ▶); program(s) used to refine structure: *SHELXL97* (Sheldrick, 2008 ▶); molecular graphics: *ORTEP-3 for Windows* (Farrugia, 1997 ▶); software used to prepare material for publication: *PLATON* (Spek, 2009 ▶).

## Supplementary Material

Crystal structure: contains datablocks global, I. DOI: 10.1107/S1600536810039905/ds2061sup1.cif

Structure factors: contains datablocks I. DOI: 10.1107/S1600536810039905/ds2061Isup2.hkl

Additional supplementary materials:  crystallographic information; 3D view; checkCIF report

## Figures and Tables

**Fig. 1.**
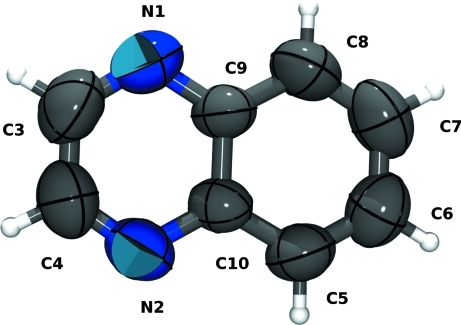
View of the title compound with the atom numbering. Displacement ellipsoids for non-H atoms are drawn at the 50% probability level.

## supplementary crystallographic information

### Experimental

For in situ crystallization, liquid quinoxaline was taken in a Lindemann glass capillary of 0.5 mm diameter. The Z' = 1 form of quinoxaline was obtained by sudden quenching of a capillary, kept in a hot water bath at 70 ^o^C, down to liquid N_2_ temperature.The capillary was aligned on a Bruker AXS Smart Apex diffractometer and data was collected at 270 K under a liquid N_2_ flow using the OXFORD N_2_ cryosystems appratus.

### Refinement

A crystal domain for the *Z'* = 1 structure was selected and indexed using the RLATT software and refined using SHELXL97. MERG 3 command was used for merging the Friedel pairs. Flack parameter was not reported as compound is achiral.

### Crystal data

**Table:**

C_8_H_6_N_2_	*D*_x_ = 1.295 Mg m^−^^3^
*M**_r_* = 130.15	Melting point = 301–305 K
Orthorhombic, *P*2_1_2_1_2_1_	Mo *K*α radiation, λ = 0.71073 Å
Hall symbol: P 2ac 2ab	Cell parameters from 734 reflections
*a* = 4.0212 (13) Å	θ = 1.8–26.0°
*b* = 7.187 (2) Å	µ = 0.08 mm^−^^1^
*c* = 23.095 (7) Å	*T* = 270 K
*V* = 667.5 (3) Å^3^	Block, pink
*Z* = 4	0.40 × 0.30 × 0.30 mm
*F*(000) = 272	

### Data collection

**Table:**

Bruker SMART CCD area-detector diffractometer	956 independent reflections
Radiation source: fine-focus sealed tube	494 reflections with *I* > 2σ(*I*)
graphite	*R*_int_ = 0.045
φ and ω scans	θ_max_ = 27.9°, θ_min_ = 1.8°
Absorption correction: multi-scan (*SADABS*; Sheldrick, 1996)	*h* = −5→5
*T*_min_ = 0.968, *T*_max_ = 0.976	*k* = −9→9
7556 measured reflections	*l* = −29→29

### Refinement

**Table:**

Refinement on *F*^2^	Primary atom site location: structure-invariant direct methods
Least-squares matrix: full	Secondary atom site location: difference Fourier map
*R*[*F*^2^ > 2σ(*F*^2^)] = 0.038	Hydrogen site location: inferred from neighbouring sites
*wR*(*F*^2^) = 0.106	H-atom parameters constrained
*S* = 0.90	*w* = 1/[σ^2^(*F*_o_^2^) + (0.0652*P*)^2^] where *P* = (*F*_o_^2^ + 2*F*_c_^2^)/3
956 reflections	(Δ/σ)_max_ < 0.001
91 parameters	Δρ_max_ = 0.13 e Å^−^^3^
0 restraints	Δρ_min_ = −0.15 e Å^−^^3^

### Special details

**Table:**

Geometry. Bond distances, angles *etc*. have been calculated using the rounded fractional coordinates. All su's are estimated from the variances of the (full) variance-covariance matrix. The cell e.s.d.'s are taken into account in the estimation of distances, angles and torsion angles
Refinement. Refinement on *F*^2^ for ALL reflections except those flagged by the user for potential systematic errors. Weighted *R*-factors *wR* and all goodnesses of fit *S* are based on *F*^2^, conventional *R*-factors *R* are based on *F*, with *F* set to zero for negative *F*^2^. The observed criterion of *F*^2^ > σ(*F*^2^) is used only for calculating -*R*-factor-obs *etc*. and is not relevant to the choice of reflections for refinement. *R*-factors based on *F*^2^ are statistically about twice as large as those based on *F*, and *R*-factors based on ALL data will be even larger.

### Fractional atomic coordinates and isotropic or equivalent isotropic displacement parameters (Å^2^)

**Table:**

	*x*	*y*	*z*	*U*_iso_*/*U*_eq_	
N1	0.2099 (7)	0.1700 (3)	0.11405 (9)	0.1298 (9)	
N2	0.2375 (6)	0.4337 (3)	0.20264 (7)	0.1131 (8)	
C3	0.3379 (8)	0.1356 (3)	0.16499 (13)	0.1287 (13)	
C4	0.3497 (7)	0.2654 (4)	0.20858 (9)	0.1197 (10)	
C5	−0.0218 (7)	0.6547 (3)	0.13897 (10)	0.1051 (9)	
C6	−0.1499 (7)	0.6977 (3)	0.08739 (11)	0.1121 (10)	
C7	−0.1662 (7)	0.5672 (4)	0.04429 (10)	0.1201 (10)	
C8	−0.0500 (9)	0.3930 (4)	0.05303 (8)	0.1210 (10)	
C9	0.0873 (6)	0.3450 (2)	0.10587 (8)	0.0900 (8)	
C10	0.1023 (6)	0.4767 (3)	0.14993 (7)	0.0842 (7)	
H3	0.42490	0.01780	0.17200	0.1550*	
H4	0.44230	0.23130	0.24390	0.1440*	
H5	−0.01510	0.74440	0.16800	0.1260*	
H6	−0.22890	0.81740	0.08070	0.1340*	
H7	−0.25820	0.59880	0.00870	0.1440*	
H8	−0.06220	0.30540	0.02350	0.1450*	

### Atomic displacement parameters (Å^2^)

**Table:**

	*U*^11^	*U*^22^	*U*^33^	*U*^12^	*U*^13^	*U*^23^
N1	0.175 (2)	0.0880 (13)	0.1265 (15)	0.0079 (13)	−0.0063 (15)	−0.0159 (10)
N2	0.1395 (18)	0.1161 (14)	0.0837 (11)	−0.0085 (13)	−0.0069 (11)	−0.0063 (9)
C3	0.156 (3)	0.0884 (14)	0.1418 (19)	0.0102 (16)	0.003 (2)	0.0182 (15)
C4	0.134 (2)	0.1240 (18)	0.1012 (14)	0.002 (2)	−0.0040 (16)	0.0271 (14)
C5	0.131 (2)	0.0819 (13)	0.1025 (14)	0.0005 (13)	0.0090 (14)	−0.0123 (10)
C6	0.1148 (19)	0.0976 (14)	0.1238 (17)	0.0038 (14)	0.0150 (17)	0.0233 (14)
C7	0.120 (2)	0.154 (2)	0.0862 (13)	−0.0023 (19)	−0.0026 (14)	0.0241 (16)
C8	0.160 (2)	0.1223 (17)	0.0806 (13)	0.0018 (19)	−0.0101 (16)	−0.0157 (12)
C9	0.1152 (17)	0.0750 (10)	0.0797 (11)	−0.0063 (12)	0.0067 (12)	−0.0083 (8)
C10	0.1008 (16)	0.0802 (10)	0.0717 (10)	−0.0131 (12)	0.0090 (10)	−0.0034 (8)

### Geometric parameters (Å, °)

**Table:**

N1—C3	1.308 (4)	C8—C9	1.383 (3)
N1—C9	1.364 (3)	C9—C10	1.391 (3)
N2—C4	1.298 (4)	C3—H3	0.9300
N2—C10	1.369 (3)	C4—H4	0.9300
C3—C4	1.373 (4)	C5—H5	0.9300
C5—C6	1.334 (4)	C6—H6	0.9300
C5—C10	1.396 (3)	C7—H7	0.9300
C6—C7	1.369 (4)	C8—H8	0.9300
C7—C8	1.352 (4)		
			
N1···N2	2.791 (3)	H4···N2^v^	2.7900
N2···N1	2.791 (3)	H7···C6^vi^	3.0900
N2···H4^i^	2.7900	H8···C8^iii^	3.0000
C6···H7^ii^	3.0900	H8···C8^iv^	3.0700
C8···H8^iii^	3.0700	H8···H8^iii^	2.4200
C8···H8^iv^	3.0000	H8···H8^iv^	2.4200
			
C3—N1—C9	116.2 (2)	C5—C10—C9	118.38 (18)
C4—N2—C10	116.26 (19)	N1—C3—H3	118.00
N1—C3—C4	123.0 (2)	C4—C3—H3	119.00
N2—C4—C3	122.9 (2)	N2—C4—H4	119.00
C6—C5—C10	120.8 (2)	C3—C4—H4	119.00
C5—C6—C7	120.6 (2)	C6—C5—H5	120.00
C6—C7—C8	120.6 (2)	C10—C5—H5	120.00
C7—C8—C9	120.1 (2)	C5—C6—H6	120.00
N1—C9—C8	119.77 (19)	C7—C6—H6	120.00
N1—C9—C10	120.69 (19)	C6—C7—H7	120.00
C8—C9—C10	119.54 (18)	C8—C7—H7	120.00
N2—C10—C5	120.67 (19)	C7—C8—H8	120.00
N2—C10—C9	121.0 (2)	C9—C8—H8	120.00
			
C9—N1—C3—C4	0.3 (4)	C10—C5—C6—C7	0.8 (4)
C3—N1—C9—C10	0.3 (4)	C5—C6—C7—C8	−0.6 (4)
C3—N1—C9—C8	179.5 (3)	C6—C7—C8—C9	−0.1 (5)
C10—N2—C4—C3	0.3 (4)	C7—C8—C9—C10	0.5 (4)
C4—N2—C10—C5	−179.3 (2)	C7—C8—C9—N1	−178.8 (3)
C4—N2—C10—C9	0.3 (4)	N1—C9—C10—N2	−0.6 (4)
N1—C3—C4—N2	−0.7 (5)	C8—C9—C10—C5	−0.3 (4)
C6—C5—C10—C9	−0.4 (4)	N1—C9—C10—C5	179.0 (2)
C6—C5—C10—N2	179.2 (3)	C8—C9—C10—N2	−179.8 (3)

Symmetry codes: (i) −*x*+1, *y*+1/2, −*z*+1/2; (ii) *x*+1/2, −*y*+3/2, −*z*; (iii) *x*−1/2, −*y*+1/2, −*z*; (iv) *x*+1/2, −*y*+1/2, −*z*; (v) −*x*+1, *y*−1/2, −*z*+1/2; (vi) *x*−1/2, −*y*+3/2, −*z*.
